# Shifts in Host Mucosal Innate Immune Function Are Associated with Ruminal Microbial Succession in Supplemental Feeding and Grazing Goats at Different Ages

**DOI:** 10.3389/fmicb.2017.01655

**Published:** 2017-08-30

**Authors:** Jinzhen Jiao, Chuanshe Zhou, L. L. Guan, C. S. McSweeney, Shaoxun Tang, Min Wang, Zhiliang Tan

**Affiliations:** ^1^Key Laboratory for Agro-ecological Processes in Subtropical Region, Hunan Research Center of Livestock and Poultry Sciences, South Central Experimental Station of Animal Nutrition and Feed Science in the Ministry of Agriculture, Institute of Subtropical Agriculture, The Chinese Academy of Sciences Changsha, China; ^2^Hunan Co-Innovation Center of Animal Production Safety Changsha, China; ^3^Department of Agricultural, Food and Nutritional Sciences, University of Alberta, Edmonton AB, Canada; ^4^CSIRO, Agriculture and Food, Queensland Bioscience Precinct, St Lucia QLD, Australia

**Keywords:** rumen, microbiota, mucosal, innate immune function, animal development process, feeding type

## Abstract

Gastrointestinal microbiota may play an important role in regulating host mucosal innate immune function. This study was conducted to test the hypothesis that age (non-rumination, transition and rumination) and feeding type [Supplemental feeding (S) vs. Grazing (G)] could alter ruminal microbial diversity and maturation of host mucosal innate immune system in goat kids. MiSeq sequencing was applied to investigate ruminal microbial composition and diversity, and RT-PCR was used to test expression of immune-related genes in ruminal mucosa. Results showed that higher (*P* < 0.05) relative abundances of *Prevotella, Butyrivibrio, Pseudobutyrivibrio, Methanobrevibacter.gottschalkii, Neocallimastix, Anoplodinium–Diplodinium*, and *Polyplastron*, and lower relative abundance of *Methanosphaera* (*P* = 0.042) were detected in the rumen of S kids when compared to those in G kids. The expression of genes encoding *TLRs, IL1α, IL1β* and *TICAM2* was down-regulated (*P <* 0.01), while expression of genes encoding tight junction proteins was up-regulated (*P <* 0.05) in the ruminal mucosa of S kids when compared to that in G kids. Moreover, irrespective of feeding type, relative abundances of ruminal *Prevotella, Fibrobacter, Ruminococcus, Butyrivibrio, Methanobrevibacter, Neocallimastix*, and *Entodinium* increased with age. The expression of most genes encoding *TLRs* and cytokines increased (*P <* 0.05) from day 0 to 7, while expression of genes encoding tight junction proteins declined with age (*P <* 0.05). This study revealed that the composition of each microbial domain changed as animals grew, and these changes might be associated with variations in host mucosal innate immune function. Moreover, supplementing goat kids with concentrate could modulate ruminal microbial composition, enhance barrier function and decrease local inflammation. The findings provide useful information in interpreting microbiota and host interactions, and developing nutritional strategies to improve the productivity and health of rumen during early life.

## Introduction

Ruminants have a remarkable ability to convert human-indigestible plant biomass into human-digestible food products through rumen microbial fermentation (Jami et al., 2013). Bacteria, fungi and protozoa decompose fibrous plant polymers into short-chain fatty acids, and release hydrogen (Kittelmann and Janssen, 2011; Kittelmann et al., 2012; Jami et al., 2013). Methanogenic archaea can scavenge hydrogen and produce methane (Janssen and Kirs, 2008). During early life, ruminal bacterial populations underwent dynamic changes at both density (Jiao et al., 2015b) and diversity (Jami et al., 2013; Rey et al., 2014) levels as the animals matured. A recent study by Dill-McFarland et al. (2017) revealed succession in colonization of the rumen by the three microbial domains (bacteria, fungi and archaea) at weaning (8 weeks old) and post-weaning (1 and 2 years old) in dairy cows.

The rumen fermentation products are mainly absorbed by the ruminal epithelial wall, and utilized by the host (Graham and Simmons, 2005). In addition to the nutrient uptake, the ruminal epithelium plays a major role in host mucosal innate immune function. The squamous rumen epithelium is comprised of four distinct strata; stratum corneum, granulosum, spinosum and basale layers (Graham and Simmons, 2005). The multicellular structures are connected through junctional complexes, thus forming the primary barrier function (Matter and Balda, 2003). In addition, rumen epithelial cells interact with lymphocytes and dendritic cells in lamina propria to maintain immune homeostasis (Trevisi et al., 2014). In goats, a high-grain diet (65% grain) could cause massive disruption of ruminal epithelial tight junctions when compared to the control diet (0% grain) (Liu et al., 2013). Despite this, a systematic characterization about how rumen mucosal innate immune function developed during early life is still not well-defined.

It is well established that the gastrointestinal commensal bacteria, such as *Bacteroides fragilis* are capable of activating and modulating immune responses in the host (Troy and Kasper, 2010). For example, Toll like receptors (*TLRs*) on the host mucosa can recognize conserved microbial domains, and trigger host innate immune responses (Akira et al., 2001; Kawai and Akira, 2010). In the rumen of dairy calves and goats, *TLR* expression was diet dependent and correlated with bacterial diversity (Chen et al., 2012; Malmuthuge et al., 2013; Liu et al., 2015), suggesting ruminal microbiota may play a role to interact with host mucosa which can mount an immune response. Fungi and protozoa can also encounter with *TLR* signaling pathway to maintain host–microbe homeostasis (Gazzinelli and Denkers, 2006; Bourgeois and Kuchler, 2012). However, the mechanism by which the ruminal microbiota interact with host mucosal innate immune system is relatively unexplored.

To address this knowledge gap, we hypothesized that age (non-rumination, transition and rumination phases) and feeding type [supplemental feeding, (S) and grazing (G)] could modulate ruminal microbiota, and further manipulate host mucosal innate immune function. To test this hypothesis, we applied a combination of MiSeq sequencing and relative RT-PCR analysis to investigate microbial diversity, as well as expression of genes encoding *TLRs*, inflammatory cytokines, and toll-IL-1 receptor (*TIR*)-domain containing adaptors.

## Materials and Methods

### Animals, Diets and Experimental Design

All management and experimental procedures were approved by the Animal Care Committee, Institute of Subtropical Agriculture, the Chinese Academy of Sciences, Changsha, China.

Thirty-two Liuyang black goat kids were separated from their dams after birth, and maintained within individual pens for the duration of the study. From birth to day 20, kids consumed goat milk and 4 kids were slaughtered at day 0 and 7 (non-rumination phase), respectively. The remaining 24 kids were randomly allocated to two groups based on different feeding types, supplemental feeding (S) and grazing (G). Between day 20 and 40, kids in S group consumed milk, forage plus concentrate, while kids in G group consumed milk and forage. The animals were weaned at day 40. From day 40 to 70, S kids were provided with concentrate and forage, while G kids grazed 8 h on pasture daily, and returned to pens overnight without receiving any extra feed. In both groups, four kids were slaughtered at each of the following ages: day 28, 42 (transition phase) and day 70 (rumination phase). All kids had free access to water. Detailed feeding management, and ingredients of concentrate and forage have been described in our previous parallel study (Jiao et al., 2015b).

### Sampling Procedures

After the kids were slaughtered, ruminal digesta samples were collected from five locations (anterior dorsal, anterior ventral, medium ventral, posterior dorsal, and posterior ventral locations) within the rumen (∼10 g each). The samples were composited, and aliquots were stored at -80°C for subsequent DNA extraction and sequencing analysis. Ruminal mucosa samples were collected from the same sites where digesta samples were collected, and rinsed three times with sterile phosphate buffered saline (PBS, pH = 7.4). Afterward, the mucosa samples were separated from the underlying muscular layer, cut into 3 mm × 3 mm fragments, composited, frozen in liquid nitrogen and stored at -80°C for later molecular analysis.

### DNA Extraction, PCR Amplification, MiSeq Sequencing and Data Analysis

Genomic DNA was extracted using the QIAamp DNA Stool Mini kit (Qiagen GmbH, Hilden, Germany) and quantified using NanoDrop ND1000 (NanoDrop Technologies, Wilmington, DE, United States). Conventional PCR was conducted to amplify bacterial V2–V3 and archaeal V6–V8 regions of the 16S rRNA genes, as well as fungal V3–V5 and protozoal V4–V5 regions of the 18S rRNA genes using primers detailed in Supplementary Table S1. The PCR conditions were as follows: pre-denaturation at 94°C for 3 min, 35 cycles of denaturation at 94°C for 30 s, annealing at 58°C for bacteria (62°C for archaea, 58°C for fungi and 54°C for protozoa) for 30 s, and elongation at 72°C for 45 s, and following with post-elongation at 72°C for 10 min. The PCR amplification products were purified using Wizard^®^ SV Gel and PCR Clean-Up System (Promega, Madison, WI, United States). Barcoded amplicons were mixed at equal molar rations, and submitted for 300 bp pair-end sequencing on an Illumina MiSeq PE300 instrument (Illumina, San Diego, CA, United States).

Raw data were filtered through Quantitative Insight into Microbial Ecology (QIIME) (Caporaso et al., 2010b) and Mothur (Schloss et al., 2009). Reads were deconvoluted into individual samples based on their barcodes. The pair-end reads were assembled based on their overlapped sequences using COPE (Connecting Overlapped Pair-End) (Liu et al., 2012) and trimmed of primers.

For bacteria and archaea, the assembled sequences were assigned to operational taxonomic units (OTUs) at a 97% identity threshold using UPARSE (Edgar, 2013). Chimeric sequences were removed using UCHIME. Taxonomy classifications were assigned against the latest Greengenes database (May 2013 release) for bacteria (DeSantis et al., 2006) and RIM-DB version13_11_13 for archaea (Seedorf et al., 2014), respectively. The taxonomic assignment was performed using Mothur based implementation of the RDP Bayesian classifier with a 0.80 confidence threshold. For fungi and protozoa, sequences were assigned into OTUs using UCLUST at a 97% identity threshold. The OTUs were classified using BLAST against SILVA database for fungi and the reference database constructed by Kittelmann and Janssen (2011) for protozoa. Sequences with identity scores, between 95 and 97% were resolved at the genus level, 90 and 95% at the family level, 85 and 90% at the order level, 80 and 85% at the class level, and 77 and 80% at the phyla level (Hristov et al., 2012). Sequences were aligned by PyNAST (Caporaso et al., 2010a), and phylogenetic trees were constructed using FastTree.

Alpha diversity was examined using the alpha rarefaction pipeline of QIIME. Principle coordinate analysis (PCoA) was performed using unweighted UniFrac distance (jackknifed beta diversity from resampling 100 times at a depth of 75% of least number of sequences). Transformation-based principal component analysis (tb-PCA) was used to ordinate the samples based on OTU abundance data using the Hellinger distance (Legendre and Gallagher, 2001), and only the top 10 OTUs were presented.

### RNA Extraction and Gene Expression

Total RNA was extracted from mucosa samples (200 mg) using TRIzol reagent (Invitrogen, Carlsbad, CA, United States). The RNA integrity was evaluated using 1% agarose gel electrophoresis, and its quantity was measured using ND1000 spectrophotometer (NanoDrop Technologies, Wilmington, DE, United States). The RNA was reversely transcribed using the First Strand cDNA Synthesis Kit (Toyobo, Osaka, Japan), and the cDNA samples were stored at -20°C until use. The RT-PCR analysis was used to evaluate expression of genes encoding *TLRs*, inflammatory cytokines, toll-IL-1 receptor (*TIR*)-domain containing adaptors, and tight junction proteins (*TJs*), with GADPH and β-actin as the housekeeping genes. The primers are listed in Supplementary Table S2, and procedures are detailed in Supplementary Text 1.

### Statistics Analysis

The effect of feeding type was examined from day 28 to 70. Data were analyzed using the MIXED procedures of SAS 8.2 (SAS Institute Inc., Cary, North Carolina, United States). Feeding type, age, and feeding type × age interaction were regarded as fixed effects, animal nested within feeding type × age was regarded as the random effect, and each individual animal was the experimental unit. The following model was used:

$${\text{Y}}_{\text{ijk}}\;=\;\mu \;+\;{\text{A}}_{\text{i}}\;+\;{\text{F}}_{\text{j}}\;+\;{\text{A}}_{\text{i}}\;\times \;{\text{F}}_{\text{j}}\;+\;{\text{Ani}}_{\text{k}}\;+\;{\text{e}}_{\text{ijk}}$$

where Y_ijk_ = dependent variable, μ = overall mean, A_i_ = fixed effect of age, F_j_ = fixed effect of feeding type, Ani_k_ = random effect of animal nested within feeding type × age, and e_ijk_ = the random residual error.

The effect of age on ruminal microbial diversity and expression of genes involved in host mucosal innate immune function was evaluated from day 0 to 70. The MIXED procedure of SAS and Tukey’s test to compare least squares means were used, with age as the fixed effect, animal within age as random effect, and individual animal as the experimental unit. The following model was used:

$${\text{Y}}_{\text{ij}}\;=\;\mu \;+\;{\text{A}}_{\text{i}}\;+\;{\text{Ani}}_{\text{j}}\;+\;{\text{e}}_{\text{ij}}$$

where Y_ij_ = dependent variable, μ = overall mean, A_i_ = fixed effect of age, Ani_j_ = random effect of animal within age, and e_ij_ = the random residual error. Orthogonal contrasts were performed to test linear and quadratic effects of age. If feeding type × age interaction was not significant within day 28 to 70, effect of age was assessed using combined data of both groups; otherwise, they were presented separately. All data were presented as least squares means, and significance was declared with *P* < 0.05.

The PROC CORR procedure of SAS was used to examine the Spearman’s rank correlations between ruminal microbial abundance and expression of mucosal innate immune function related genes. Only those correlation coefficients above 0.50, and *P* < 0.05 were defined as correlated with each other and plotted using corrplot package in the R studio.

### Nucleotide Sequence Accession Numbers

Sequencing data in this study were deposited in the NCBI Sequence Read Archive (SRA) under accession numbers PRJNA362795.

## Results

### OTU Diversity and Similarity Analysis

**FIGURE 1:**
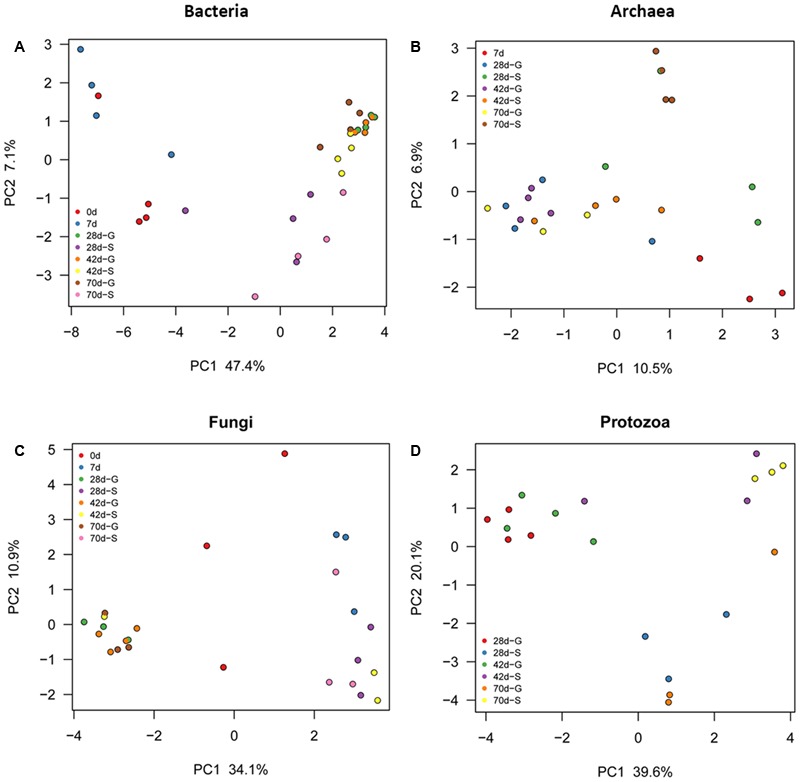
Principal coordinate analysis (PCoA) of each ruminal microbial domain in S and G kids at different ages. **(A)** Bacteria, **(B)** Archaea, and **(C)** Fungi, and **(D)** Protozoa.

**FIGURE 2:**
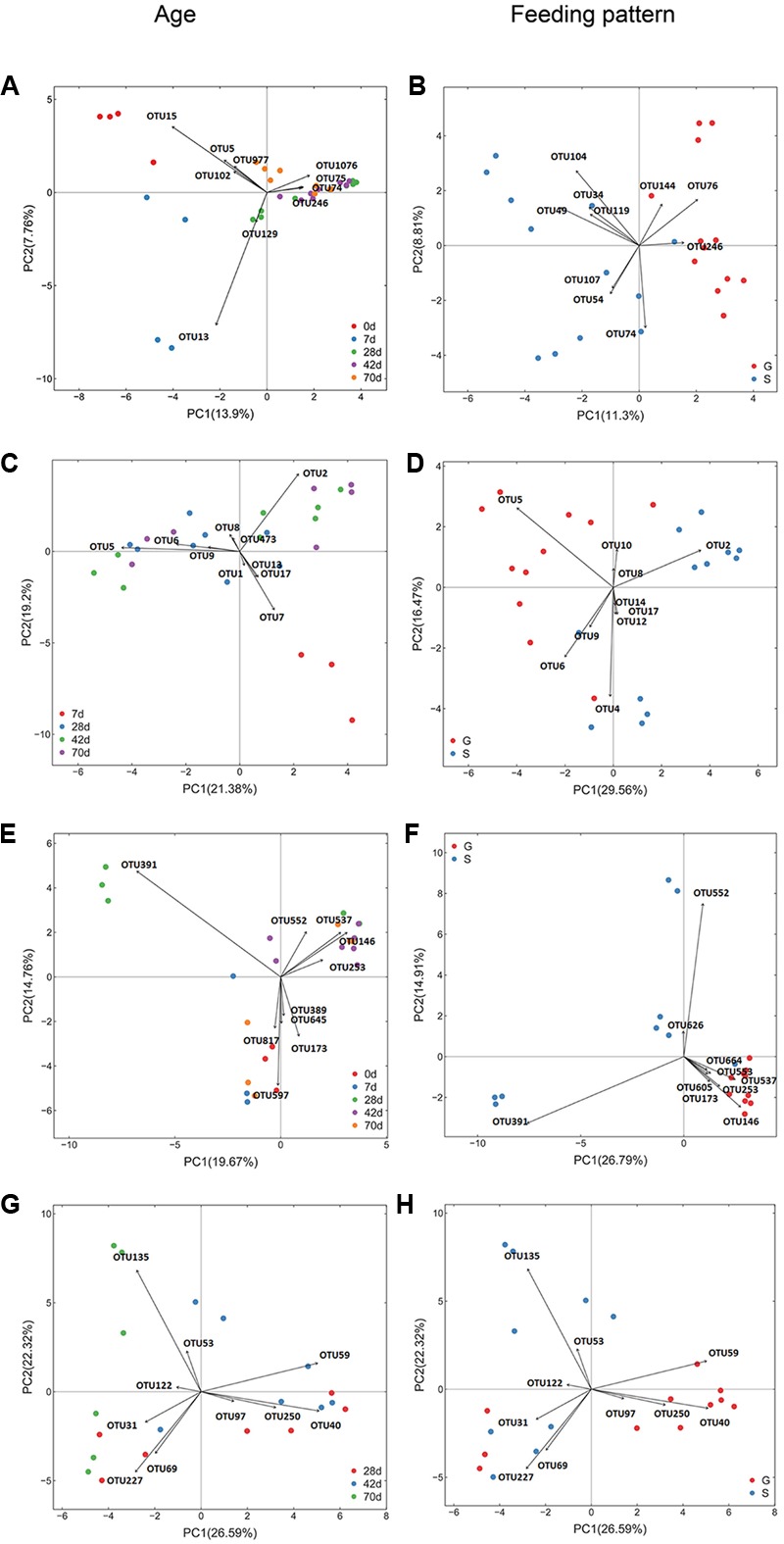
Transform-based PCA of each ruminal microbial domain for separation of different ages and feeding types in kids. The top 10 OTUs contributing to the separation of samples were displayed on the figure (OTU number). **(A)** Bacterial OTUs for discrimination of different ages, **(B)** Bacterial OTUs for discrimination of different feeding types. For bacteria **(A,B)**, OTU15, *Arcobacter* genus; OTU5, *Escherichia* genus; OUT977, *Escherichia* genus; OTU102, *Pasteurellaceae* family; OTU13, *Halomonadaceae* family; OTU129, *Delftia* genus; OTU74, *Prevotella* genus; OTU75, *Lachnospiraceae* family; OTU1076, *Prevotella* genus; OTU246, *Prevotella* genus; OTU76, *Prevotella* genus; OTU144, *Prevotella* genus; OTU104, *Succinivibrionaceae* family; OTU34, *Prevotella* genus; OTU49, *Prevotella* genus; OTU119, *Bacteroidales* order; OTU107, *Succinivibrionaceae* family; OTU54, *Ruminococcaceae* family. **(C)** Archaeal OTUs for discrimination of different ages, **(D)** Archaeal OTUs for discrimination of different feeding types. For archaea **(C,D)**, OTU5, *Methanomassiliicoccaceae* family; OTU2, *Mbb. gottschalkii*clade; OTU7, Archaea domain; OTU6, *Group12_sp._ISO4-H5*; OTU9, *Group9_sp._ISO4-G1*; OTU8, *Mbb. Ruminantium* clade; OTU4, *Methanomassiliicoccaceae* family; OTU1, *Group10_sp.*; OTU10, *Mbb. acididurans* clade; OTU12, *Mbb. mobile* clade; OTU13, *Euryarchaeota* phylum; OTU14, *Euryarchaeota* phylum; OTU17, Archaea domain; OTU473, *Mbb. Ruminantium* clade. **(E)** Fungal OTUs for discrimination of different ages, **(F)** Fungal OTUs for discrimination of different feeding types. For fungi **(E,F)**, OTU391, *Neocallimastix* genus; OTU579, *Saccharomyces* genus; OTU146, *Uwebraunia* genus; OTU537, *Piromyces* genus;OTU173, *Boeremia* genus; OTU817, unclassified fungi; OTU645, *Eupenicillium* genus; OTU552, *Piromyces* genus; OTU253, *Knufia* genus; OTU389, Neurospora genus; OTU553, *Neocallimastix* genus; OTU664, *Microcyclosporella* genus; OTU626, *Sarocladium* genus; OTU605, *Neocallimastix*genus. **(G)** Protozoal OTUs for discrimination of different ages, **(H)** Protozoal OTUs for discrimination of different feeding types. For protozoa **(G,H)**, OTU135, *Polyplastron* genus; OTU40, *Epidinium* genus; OTU59, *Entodinium* genus; OTU227, *Entodinium* genus; OTU69, *Entodinium* genus; OTU50, *Eremoplstron-Diploplastron* genus; OTU31, *Entodinium* genus; OTU53, *Anoplodinium–Diplodinium*; OTU97, *Epidinium* genus; OTU122, *Polyplastron* genus.

As reflected by rarefaction curve, sequencing depth was adequate to cover the community of each microbial domain, with Good’s coverage ranged from 0.980 to 0.999 (**Supplementary Figure S1** and Table S3). Alpha diversity of ruminal bacteria, archaea and fungi was more abundant (*P* < 0.05) in G kids compared to that in S kids. Alpha diversity indices (OTU number, Chao, Ace and Shannon) of ruminal bacteria and archaea in both groups increased with age (*P* < 0.01). For G kids, alpha diversity indices of ruminal fungi increased (*P* < 0.01), while those indices of ruminal protozoa decreased quadratically (*P* < 0.05) with age. PCoA analysis revealed that microbial communities could be clearly discriminated by both age and feeding type (**Figure 1**). The tb-PCA results revealed the top 10 OTUs within each microbial domain contributing to separation at different ages and for feeding types (**Figure 2**). Details of these are presented in Supplementary Text 2.

### Rumen Microbial Community Changes-Bacteria

**FIGURE 3:**
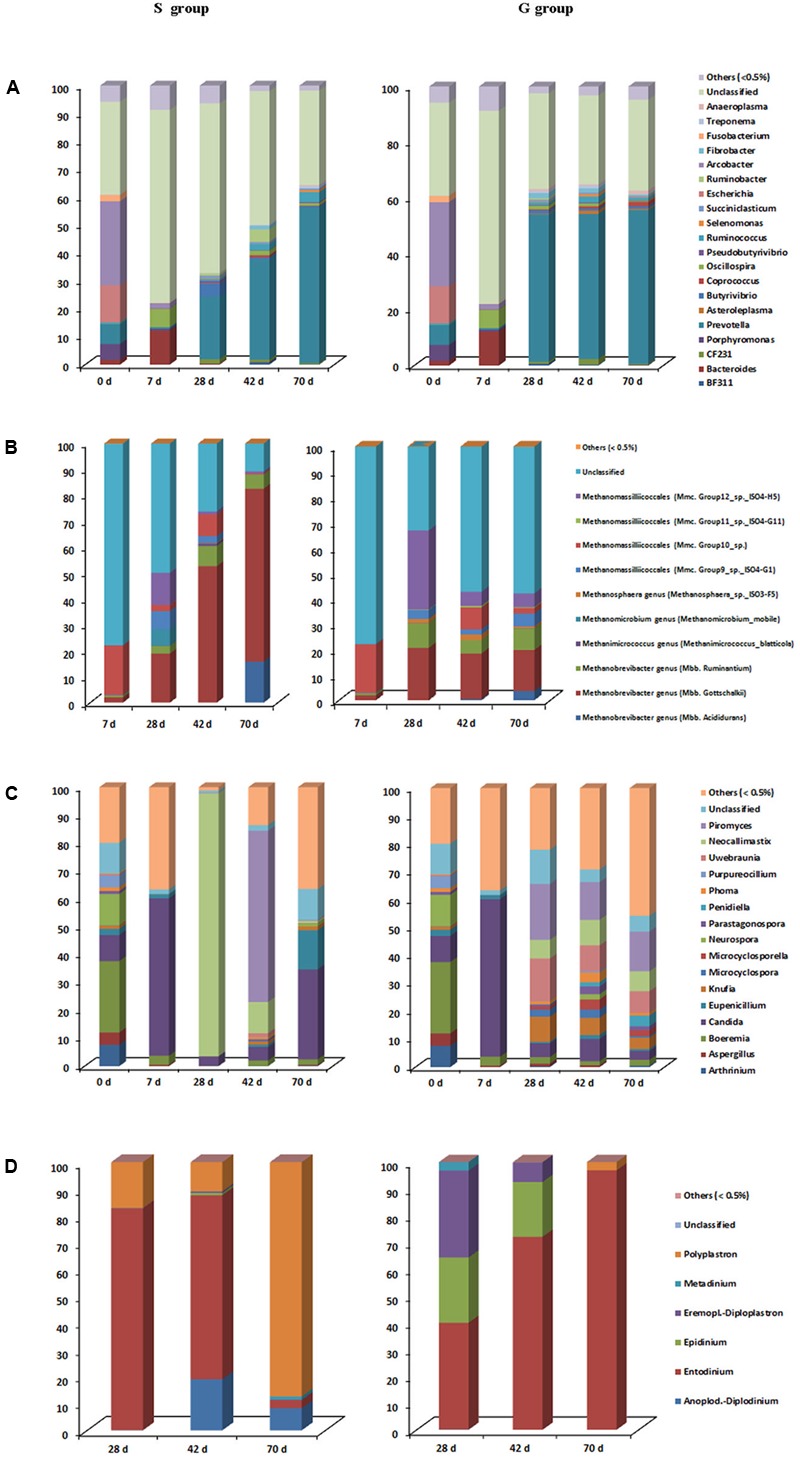
Taxonomic composition of each ruminal microbial domain in S and G kids at different ages. **(A)** Bacteria, **(B)** Archaea, **(C)** Fungi, and **(D)** Protozoa.

A total of 20 phyla and 48 genera were identified in all samples, with 23 bacterial genera each accounting for >0.5% of all sequences. A great proportion of sequences (31.2–69.2%) remained unclassified at the genus level (**Figure 3A**). Relative abundances of *Prevotella, Asteroleplasma, Coprococcus* were greater (*P* < 0.05), while relative abundances of *Butyrivibrio* and *Pseudobutyrivibrio* were lower (*P* = 0.011) in the rumen of G kids compared to those in S kids. In both groups, *Arcobacter* (30.1%), *Escherichia* (13.3%), *Porphyromonas* (5.7%) and *Fusobacterium* (2.1%) were predominant ruminal genera at day 0, whist their relative abundances declined to minimal values (<0.2%) afterward. Conversely, *Prevotella* relative abundance increased drastically (*P* < 0.01) with age, being the most dominant genus after day 28 (>20%), while *Bacteroides* relative abundance was high (12.1%) at only day 7. Moreover, relative abundances of ruminal *BF311, CF231, Butyrivibrio, Pseudobutyrivibrio*, and *Fibrobacter* in both groups increased quadratically (*P* < 0.05) with age. After day 28, relative abundances of ruminal *Asteroleplasma* and *Anaeroplasma* ranged from 0.3 to 1.2% in G kids, while their ruminal relative abundances were low (<0.2%) in S kids. Relative abundances of ruminal *Akkermansia, Corprococcus, Ruminococcus, Selenomonas, Succiniclasticum* and *Treponema* ranged from 0.1 to 3.6% in both groups. And relative abundances of *Enterococcus* and *Lactobacillus* were low (<1.0%) in the rumen of both groups.

### Rumen Microbial Community Changes-Archaea

Archaea were not detectable at day 0, and began to be detected at day 7. The relative abundance of *Mbb. gottschalkii* within the *Methanobrevibacter* genus was greater (*P* = 0.022), while relative abundances of genus *Methanosphaera*, as well as *Group 11 and 12* within the *Methanomassilliicoccales* order were lower (*P* < 0.05) in the rumen of S kids compared to those in G kids (**Figure 3B**). Within the *Methanobrevibacter* genus, relative abundances of *Mbb. gottschalkii* (16.1–66.7%) and *Mbb. ruminantium* (2.9–9.7%) clades increased drastically from day 28, constituting the top two abundant clades. The highest relative abundance of *Mbb.acididurans* in the rumen of S and G kids was 15.9% (day 70) and 3.7% (day 70), respectively. Relative abundances of *Methanimicrococcus* and *Methanosphaera* genera were low (<2.0%) in the rumen of both kids, while *Methanomicrobium* genus was only detected in the rumen of S kids at day 28 (6.4%). Furthermore, a significant proportion of *Methanomassilliicoccales* (from 12.1 to 34.8%) was found in the rumen of both groups.

### Rumen Microbial Community Changes-Fungi

Two fungal phyla, *Ascomycota* and *Neocallimastigomycota*, and 103 fungal genera were identified, with 16 fungal genera each accounting for >0.5% of all sequences (**Figure 3C**). The relative abundance of *Neocallimastix* was greater (*P* < 0.001), while relative abundances of *Microcyclosporella, Knufia, Parastagonospora* and *Uwebraunia* were lower (*P* < 0.05) in the rumen of S kids compared to those in G kids. Ruminal *Boeremia* (25.6%), *Neurospora* (11.3%), *Arthrinium* (7.6%), *Aspergillus* (4.5%), *Purpureocillium* (4.3%) were the top five abundant genera at day 0, while their relative abundances declined significantly (*P* < 0.01) afterward. The highest relative abundance of *Candida* in the rumen was 56.4% (day 7). The relative abundances of *Knufia, Microcyclospora, Microcyclosporella, Parastagonospora, Penidiella, Phoma* and *Uwebaunia* in the rumen of S kids were low (<2%) at all ages, while their relative abundances in the rumen of G kids increased with age (*P* < 0.05). Relative abundances of ruminal *Neocallimastix* and *Piromyces* in S kids increased quadratically with age (*P* < 0.05), peaking at 94.2% (day 28) and 61.3% (day 42), respectively, while their ruminal relative abundances in G kids increased linearly with age (*P* < 0.05), peaking at 7.1% (day 70) and 14.3% (day 70), respectively.

### Rumen Microbial Community Changes-Protozoa

Protozoa began to colonize after day 28, with >99% sequences classified into six genera (**Figure 3D**). The relative abundances of *Anoplodinium–Diplodinium* and *Polyplastron* were greater (*P* < 0.05), while *Polyplastron* relative abundance was lower (*P* < 0.001) in the rumen of S kids compared to those in G kids. *Entodinium* was the most abundant genera (39.9–96.8%) in the rumen, with its relative abundance declined linearly (*P* = 0.001) in S kids, while increased linearly (*P* = 0.002) in G kids from day 28 to 70. Relative abundances of *Anoplodinium–Diplodinium* and *Polyplastron* were high (8.2–87.4%) in the rumen of S kids, but low (<3.0%) in the rumen of G kids. In contrast, the relative abundances of ruminal *Epidinium* and *Eremoplstron-Diploplastron* were low (<1.0%) in S kids, but high in G kids (7.3–32.4%).

### Expression of Genes Involved in Ruminal Mucosal Innate Immune Function

**FIGURE 4:**
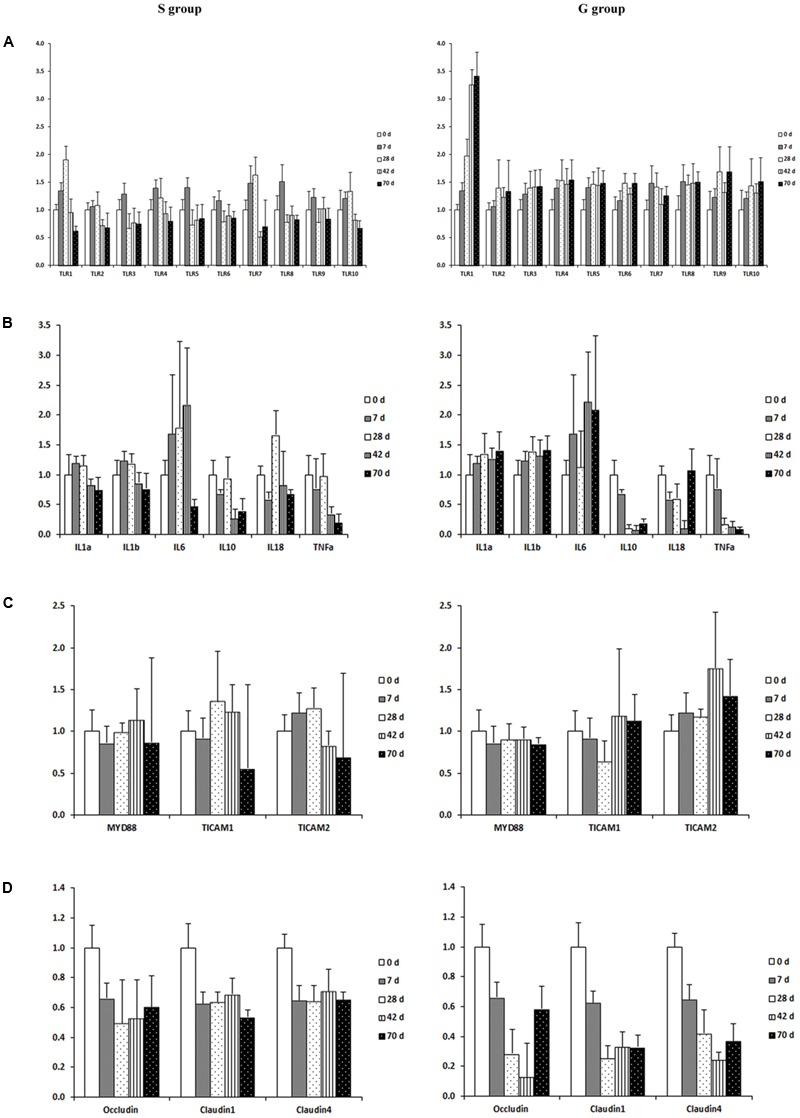
Expression of genes encoding host mucosal innate immune function in the ruminal mucosa of S and G kids at different ages. **(A)** Toll like receptors, **(B)** Cytokines; **(C)** TIR-containing adaptors; **(D)** Tight junction proteins.

The expression of genes encoding *TLRs* (1–10) was down-regulated in the ruminal mucosa of S kids when compared to that in G kids. For S kids, mRNA abundance of *TLRs1, 2, 4, 7*, and *10* increased slightly (*P* < 0.05) from day 0 to 28, and then decreased afterward (**Figure 4A**). The mRNA abundance of other *TLRs* increased during the first week (*P* < 0.05), and declined afterward. Conversely, for G kids, expression of *TLRs* increased from day 0 to 28 (*P* < 0.01), remaining relatively stable afterward.

When genes involved in cytokine production were compared, the expression of *IL1α* and *IL1β* was up-regulated (*P <* 0.01), while expression of *IL10* and *TNFα* was down-regulated (*P <* 0.01) in the ruminal mucosa of G kids when compared to those in S kids (**Figure 4B**). The mRNA abundance of *IL1α, IL1β* and *IL6* in the ruminal mucosa of S kids increased from day 0 to 28, declined afterward, while their expression in the ruminal mucosa of G kids increased (*P* < 0.05) from day 0 to d 70. In both groups, mRNA abundance of *IL10, IL18* and *TNFα* in the ruminal mucosa declined with age (*P* < 0.05).

For genes encoding *TIR* domain-containing adaptors, *MYD88* expression was age- and feeding type-independent (**Figure 4C**). The expression of *TICAM2* (*P* = 0.003) was up-regulated in the ruminal mucosa of G kids when compared to that in S kids. Age exerted a quadratic effect (*P* = 0.006) on *TICAM1* mRNA abundance in the ruminal mucosa of S kids. *TICAM2* mRNA abundance in the ruminal mucosa of S kids increased during the first month and declined afterward, whist its value in G kids increased (*P* = 0.043) from day 0 to 70.

For genes encoding tight junction proteins (*TJs*), the expression of *Occludin, Claudin1* and *Claudin4* was elevated (*P <* 0.05) in the ruminal mucosa of S kids when compared to those in G kids (**Figure 4D**). Furthermore, expression of *TJs* declined (*P* < 0.05) from day 0 to 70 in the ruminal mucosa of both groups.

### Relationship between Microbial Community and Expression of Genes Involved in Ruminal Mucosal Innate Immune Function

**FIGURE 5:**
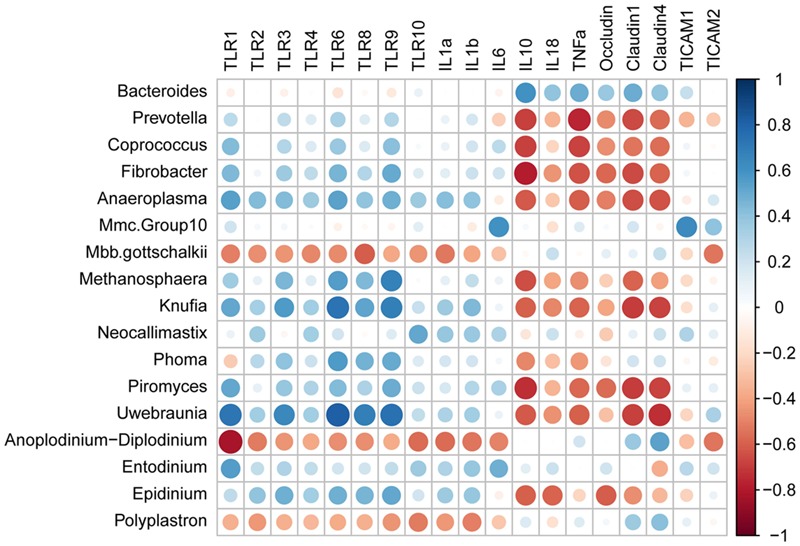
Spearman’s rank correlation coefficients between relative abundances of ruminal microbiota and expression of genes involved in host mucosal innate immune function.

As presented in **Figure 5**, *TLR1* mRNA abundance was positively correlated with (*R* > 0.50) relative abundances of *Anaeroplasma, Knufia, Piromyces, Uwebraunia*, and *Entodinium*, while negatively correlated with (*R* < -0.50) relative abundances of *Mbb. gottschalkii* and *Anoplodinium–Diplodinium*. *TLR2* mRNA abundance was negatively correlated with (*R* = -0.52) *Anoplodinium–Diplodinium* relative abundance, *TLR4* mRNA abundance was negatively correlated with (*R* = -0.50) *Mbb. gottschalkii* relative abundance, while *TLR3* mRNA abundance was positively correlated with (*R* > 0.50) relative abundances of *Knufia* and *Uwebraunia*. *TLR6* mRNA abundance was positively correlated with (*R* > 0.50) relative abundances of *Anaeroplasma, Kufia, Neocallimastix*, and *Uwebraunia*). *TLR8* mRNA abundance was positively correlated with (*R* > 0.50) relative abundances of *Kufia, Neocallimastix*, and *Uwebraunia*, while negatively correlated with (*R* = -0.61) *Mbb. gottschalkii* relative abundance. *TLR9* mRNA abundance was positively correlated with (*R* > 0.50) relative abundances of *Fibrobacter, Anaeroplasma, Kufia, Neocallimastix, Uwebraunia*, and *Epidinium*. *TLR10* mRNA abundance was positively correlated with (*R* = 0.51) *Neocallimastix* relative abundance, while negatively correlated with (*R* < -0.50) relative abundances of *Anoplodinium–Diplodinium* and *Polyplastron*. The mRNA abundance of *IL1α* and *IL1β* was negatively correlated with (*R* < -0.50) relative abundances of *Mbb. gottschalkii, Anoplodinium–Diplodinium* and *Polyplastron*. *IL6* mRNA abundance was positively correlated with (*R* = 0.60) *Mmc.Group10* relative abundance, while *IL18* mRNA abundance was negatively correlated with (*R* = -0.59) *Epidinium* relative abundance. The mRNA abundance of *IL10* and *TNFα* was positively correlated with (*R* > 0.50) *Bacteroides* relative abundance, but negatively correlated with (*R* < -0.50) relative abundances of *Bacteroides, Prevotella, Coprococcus, Fibrobacter, Anaeroplama, Methanosphaera, Phoma, Piromyces*, and *Uwebraunia*. The mRNA abundance of *Occludin, Claudin1* and *Claudin4* was negatively correlated with (*R* < -0.50) relative abundances of most microbes. *TICAM1* mRNA abundance was positively correlated with (*R* = 0.63) *Mmc.Group10* relative abundance, while *TICAM2* mRNA abundance was negatively correlated with (*R* < -0.50) relative abundances of *Mbb. gottschalkii* and *Anoplodinium–Diplodinium*.

## Discussion

The goats underwent a drastic change in nutrient supply from a high-fat milk diet during non-rumination stage to a forage and concentrate based diet during rumination stage (Jiao et al., 2015b). Therefore, the observed shifts in both microbial density (Jiao et al., 2015b) and microbial composition in the rumen could be driven by the interaction between dietary changes and animal growth (ages). Meanwhile, the observed associations between the abundance of certain microbial taxa and expression of genes encoding *TLRs* and *TJ* proteins suggest that shifts in microbiota may influence the development of ruminal mucosal innate immune function. Historically, rumen epithelial wall has been considered “immune intact” for its tolerance in highly dynamic and abundant microbiota. However, recent studies have highlighted its innate immune function for the consistently detectable expression of genes encoding *TLR*s and *TJ* proteins in dairy and beef cattle (Devant et al., 2016; McCann et al., 2016), and the differences in the expression of these genes have been related to the ruminal microbial population changes (Chen et al., 2012). It is known that the rumen only develops after animals start to consume solid feed, and the undeveloped rumen epithelial structure lacks of distinct corneum, granulosum, spinosum, and basale strata (Graham and Simmons, 2005). This suggests that during early life the ruminal epithelial cells may trigger innate immune responses through molecular recognition pattern mechanism (such as *TLRs*) similar to the identified mechanism in the intestinal tissues of monogastric animals.

Indeed, the elevated expression of *TLRs* was detected in the rumen of kids during the first week of life. It has been reported that the elevated expression of *TLRs* is associated with cytokine production (Zeytun et al., 2010). Although the production of cytokines was not measured in this study, the enhanced expression of genes encoding pro-inflammatory cytokines, *IL1α, IL1β* and *IL6* may indirectly support the higher cytokine production which could be led by the *TLRs*. On the other hand, the decreased gene expression of *TJ* proteins suggests higher mucosal permeability during the first week (Matter and Balda, 2003). Malmuthuge et al. (2013) reported the intake of a calf starter ration decreased expression of *TJ* genes in the small intestine of 8 weeks calves, and speculated the increased gut permeability could be associated with higher nutrient absorption capacity. Therefore, the potential higher permeability in the rumen could be also related to the nutrient absorption during early life.

In the meantime, our findings showed changes in ubiquitous microbiota, which could directly or indirectly influence ruminal innate immune function during early life. Kids were only exposed to the microbiota of their dams within 12 h of birth, after which they were separated and hand fed goat milk for 20 days which constituted the only nutrient intake. Thereby, we speculate that the microbiota detected at day 0 may be acquired from the mother at birth as well as from the environment and milk during the first day. Firstly, the predominance of *Escherichia* and *Arcobacter* at day 0 may play a role in stimulating the expression of *TLR*s, as several species in these genera, such as *E. coli, A. butzleri, A. cryaerophilus*, and *A. skirrowii* have been reported to modulate immune responses in mice (Bambou et al., 2004; Ferreira et al., 2016). Secondly, lactic acid bacteria (LAB), *Enterococcus* and *Lactobacillus* were also found in the rumen of new-born goats. Although their relative abundances were lower in the rumen compared to those in the small intestine (Jiao et al., 2016), LAB have been reported to enhance gut immune function, and consequently widely used as probiotics in both humans and animals (Walter, 2008; Franz et al., 2011). Thirdly, mucin degraders *Bacteroides* and *Akkermansia* surged in abundance during the first week. And the species, *A. muciniphila* and *B. fragilis* are potentially health-promoting bacteria which have anti-inflammatory attributes (Collado et al., 2007; Troy and Kasper, 2010). Finally, several invasive fungal pathogens, such as *Aspergillus* and *Candida* are capable of raising host–fungi interactions (Bourgeois and Kuchler, 2012), and can simulate *IL1α* production (Steele and Fidel, 2002). Their presence during the first week suggests that fungi may also play a role in development of ruminal mucosal innate immune function.

In addition to above findings on the microbial colonization and innate immune function during the first week, we observed that feeding type could impact microbial composition and expression of genes involved in innate immune function. After solid feed offered, differential expression of *TLRs* and *TJs* genes was detected between two feeding types, with lower expression of genes involved in inflammation and higher expression of genes encoding barrier function in the ruminal mucosa of S kids (detailed later). In intensive farming, supplementation of concentrate to young ruminants at a relative early age is a common practice which is considered to enhance the development of rumen (Jiao et al., 2015b). Therefore, the following discussion will mainly focus on S kids.

The expression profile for all *TLRs* was not consistent, which could reflect varied responses to the changes in the microbiota driven by the diet through the growth of S-kids. Indeed, relative abundances of bacterial members of *Prevotella* and *Fibrobacter*, archaeal members of *Mbb. gottschallkii* and *Mbb. ruminantium*, fungal members of *Neocallimastix* and *Priomyces*, protozoal members of *Entodinium* and *Epidinium* increased markedly after solid feed offered. Such changes are similar to a recent study reported by Dill-McFarland et al. (2017) who tracked ruminal microbiota from weaning (8 week) to first lactation (2 year) in the same heifers. These microbes are common members of gastrointestinal microbiota in ruminants, and are well recognized as efficient in degrading plant cellulosic fibers (Kittelmann et al., 2012; Henderson et al., 2015). Although the correlation relationship does not necessarily indicate a direct causal effect, the observed multiple significant correlations between relative abundances of these microbial taxa and expression of *TLRs* provide some insights on potential host–microbiota interaction in the rumen. Considering ruminal digesta and epithelium harbor more microbes in elder than younger ruminants (Jiao et al., 2015a,b), the down-regulated *TLR* expression after d 20 suggests a mechanism to minimize uperfluous inflammatory responses to commensal flora as proposed previously (Malmuthuge et al., 2012). Our observed down-regulated expression of genes encoding pro-inflammatory cytokines and stable expression of genes encoding *TJ* proteins confirm this speculation. Two possible reasons might contribute to the minimized immune responses. Firstly, relative abundances of butyrate-producing bacteria in ruminal digesta (*Butyrivibrio* and *Pseudobutyrivirio*) and epithelium (*Butyrivibrio* and *Campylobacter*) (Segain et al., 2000; Jiao et al., 2015a) increased, and then butyrate production was elevated (Jiao et al., 2015b). It is well established that butyrate can inhibit inflammatory responses via inhibition of NF kappa B activation (Segain et al., 2000). Secondly, relative abundances of mucin-degraders, such as *Ruminococcus* increased, and the enhanced mucus production could partly promote barrier function and alleviate inflammatory responses (Crost et al., 2013).

As described above, supplementing kids with concentrate in early life altered ruminal microbial diversity and increased expression of genes involved in barrier function. Compared to G kids, down-regulation of *TLR* signaling and pro-inflammatory cytokines revealed a potential to resist local inflammation in the ruminal mucosa of S kids (Liu et al., 2013). In general, *TLRs* can recognize microbial conserved molecular patterns, through *TIR*-domain containing adaptors such as *MYD88, TICAM1, TICAM2* and *TIRAF* (Akira et al., 2001). The lower *TICAM2* expression in the ruminal mucosa of S kids indicates that this molecule protein may play an essential role in differentiating immune responses to these two feeding types. Another explanation is the reduced permeability of the maturing rumen mucosa, as indicated by elevated expression of genes encoding *TJ* proteins in the rumen of S kids, preventing luminal microbiota entering the mucosa (Groschwitz and Hogan, 2009). The third important factor can be attributed to greater butyrate production observed in the same S kids (Jiao et al., 2015b), which is known to decrease production of pro-inflammatory cytokines (Segain et al., 2000).

On the other hand, the altered expression of genes in the ruminal mucosa of S kids could be due to the suppressed microbial diversity when compared to that in G kids. For example, increased relative abundances of *Pseudobutyrivibrio* and *Butyrivibrio* in the rumen of S kids could be associated with enhanced anti-inflammatory effects (Kopecny et al., 2003). Secondly, some species of *Methanosphera*, which have been reported to activate host dendritic cells, and induce release of inflammatory cytokines *TNFα* and *IL1β* (Bang et al., 2014), were more abundant in the rumen of G kids due to the pectins in the forages (Janssen and Kirs, 2008). Finally, *Microcyclosporella* and *Parastagonospora* are well-known fungal plant–pathogens (Frank et al., 2010; John et al., 2016), and their greater relative abundances in the rumen of G kids suggest the notion that fungal composition is diet-driven (Denman et al., 2008; Kittelmann et al., 2012), which provokes further consideration of their roles in modulating host immune function.

**FIGURE 6:**
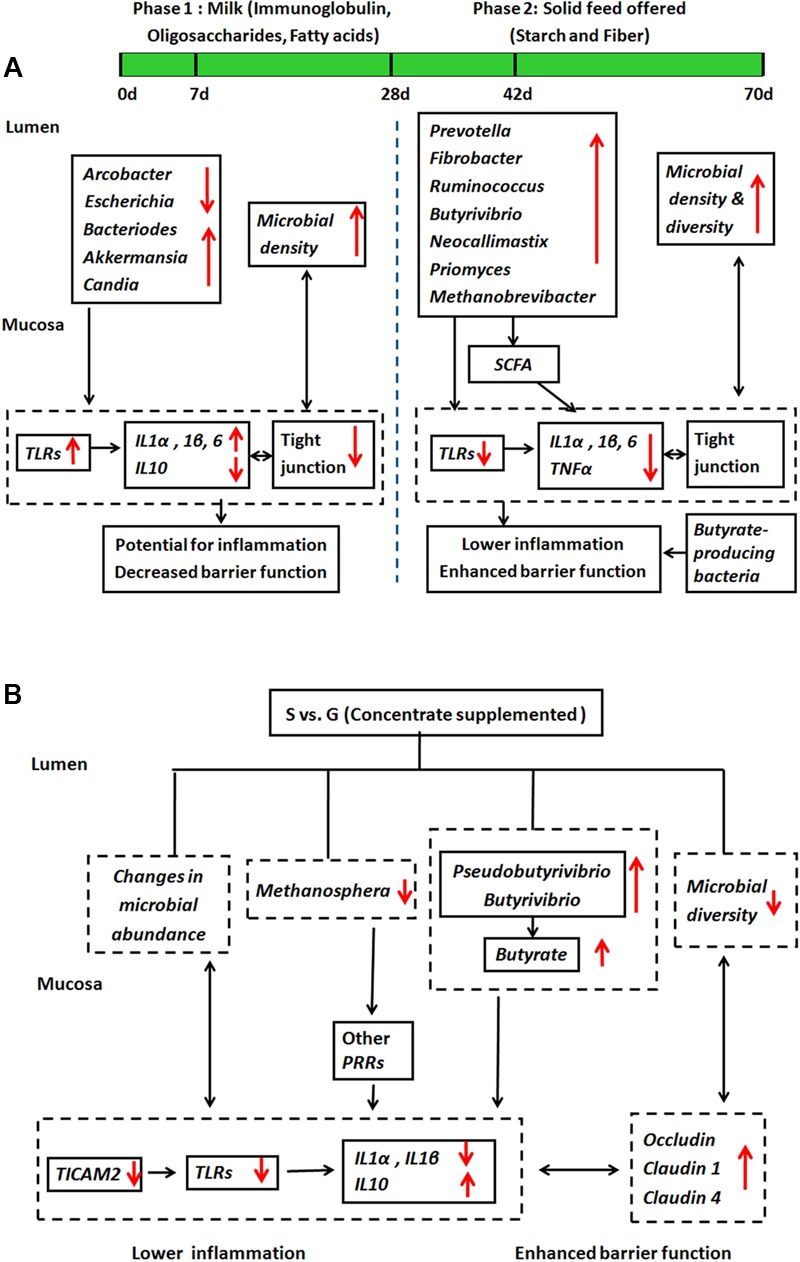
A working hypothesis of how ruminal microbiota affect host mucosal innate immune function. **(A)** During animal development in S kids, and **(B)** the effect of feeding type.

To our knowledge, limited studies research on microbiota and host innate immune function cross-talk in the rumen, and how such interaction can be impacted by dietary interventions. Based on the findings from current study, we proposed a working model for interactions between ruminal microbiota and mucosal innate immune function during animal development in S kids (**Figure 6A**) and the effect of feeding type intervention (**Figure 6B**). During the first week of life, milk nutrients facilitate colonization of mucin-degraders and fungi (*Candida*), which activate *TLR* signaling and provoke potential local inflammation. The starch and fiber alters microbial composition and diversity in ruminal digesta and mucosa, leading to enhanced SCFAs, especially butyrate production. These partly contribute to decreased inflammation by lower *TLR* signaling, and enhanced barrier function through stable *TJs* signaling, suggesting a mechanism to minimize superfluous inflammatory responses to commensal microbiota in S kids (**Figure 6A**). Furthermore, supplementing kids with concentrate alters ruminal microbial composition, diversity and products, giving rise to lower inflammation and enhanced barrier function (**Figure 6B**). Although the role of commensal microbiota on host mucosal immune function in the rumen is far from clear, our results provide some cues on the communication between ruminal microbiota and ruminal mucosal immune function that can be manipulated by dietary changes and developmental ages.

## Conclusion

Ruminal microbial diversity (bacteria, archaea, fungi and protozoa) and expression of genes involved in ruminal mucosal innate immune function was age-related in both supplemental feeding and grazing goat kids. Concentrate supplementation during the early stages of life had the potential to modulate ruminal microbial composition, enhance barrier function and decrease local inflammation. Our results provide a model for integrating information on ruminal microbial diversity and function with measures of host mucosal immunity in the maturing ruminants. Future studies are warranted to investigate the actual contribution of specific microbial species in modulating host mucosal innate immune maturation.

## Author Contributions

JJ and ZT designed the research; JJ and CZ conducted the research; ST and MW were involved in the animal experiments; JJ, LG, and CM performed the statistical analysis and contributed to the interpretation of results. JJ and LG wrote the draft of the manuscript. All authors read, approved the final manuscript, and have no competing interests.

## Conflict of Interest Statement

The authors declare that the research was conducted in the absence of any commercial or financial relationships that could be construed as a potential conflict of interest.

## Supplementary Material

FIGURE S1 Rarefaction curves of each ruminal microbial domain in S and G kids at different ages. **(A)** Bacteria, **(B)** Archaea, **(C)** Fungi, and **(D)** Protozoa.
