# Supplementary figures and images for: Shifts in Host Mucosal Innate Immune Function Are Associated with Ruminal Microbial Succession in Supplemental Feeding and Grazing Goats at Different Ages
Source: Front Microbiol. 2017 Aug 30;8:1655. doi: 10.3389/fmicb.2017.01655 (PMC5582421; doi:10.3389/fmicb.2017.01655)

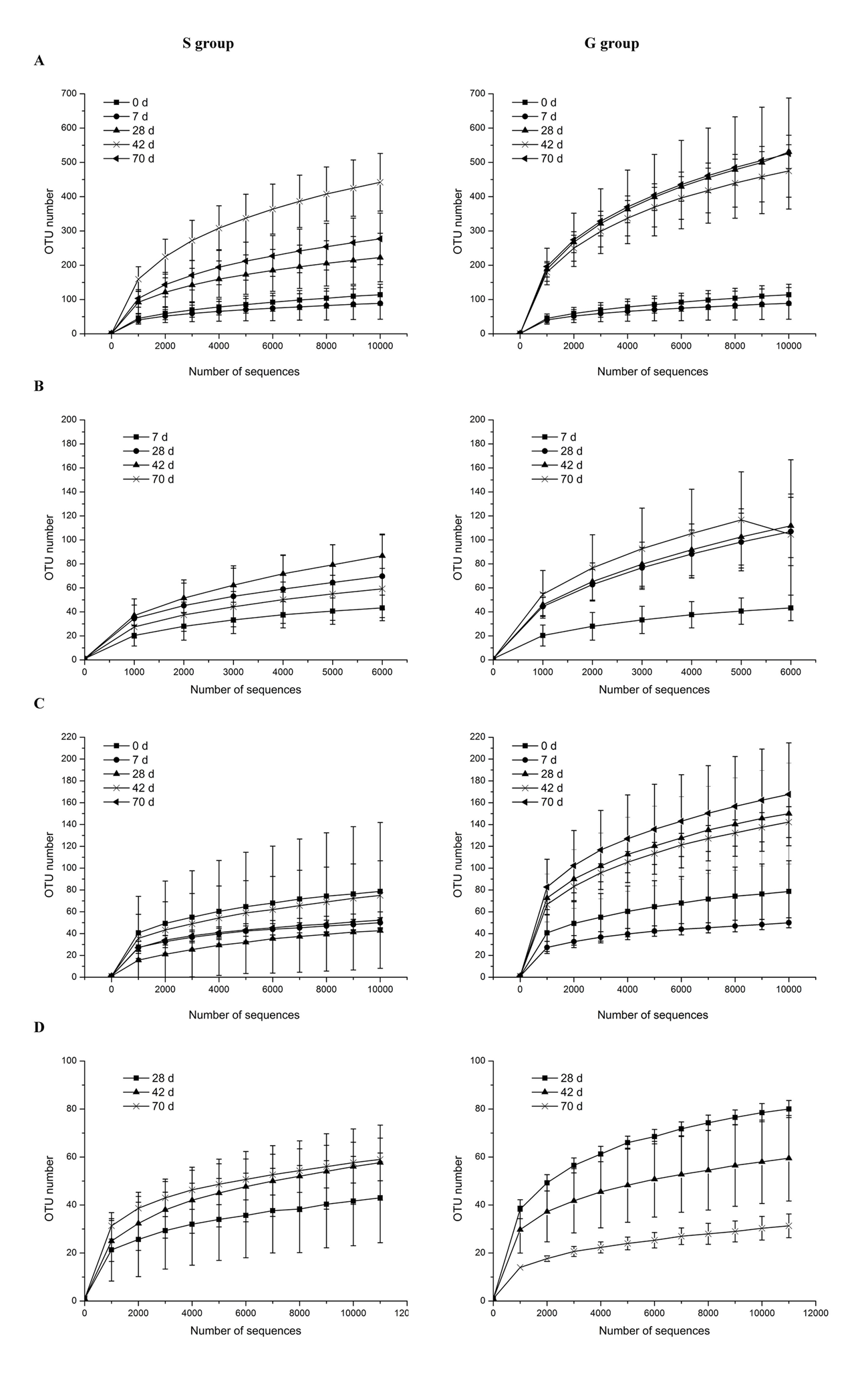

Supplement: FIGURE S1 — Rarefaction curves of each ruminal microbial domain in S and G kids at different ages. (A) Bacteria, (B) Archaea, (C) Fungi, and (D) Protozoa. [file Image_1.TIF]
