# Supplementary material for: Shifts in Host Mucosal Innate Immune Function Are Associated with Ruminal Microbial Succession in Supplemental Feeding and Grazing Goats at Different Ages
Source: Front Microbiol. 2017 Aug 30;8:1655. doi: 10.3389/fmicb.2017.01655 (PMC5582421; doi:10.3389/fmicb.2017.01655)
Supplement: Supplementary file 2 [file Table_1.docx]

**Table S1.** Primers for MiSeq sequencing

| Primer name | Sequence (5' to 3') | length (bp) | Literature cited |
| --- | --- | --- | --- |
| 104F | GGCGVACGGGTGAGTAA | 436 | Hristov et al., 2012 |
| 540R | CCGCNGCNGCTGGCAC |  |  |
| Ar915aF | AGGAATTGGCGGGGGAGCAC | 492 | Kittelmannet al., 2011 |
| Ar1386R | GCGGTGTGTGCAAGGAGC |  |  |
| Fun509F | TGGAGGGCAAGTCTGGTG | 500 | Hristovet al., 2012 |
| FunSSUR | TCGGCATAGTTTATGGTTAAG |  |  |
| RP841F | GACTAGGGATTGGAGTGG | 511 | Kittelmannet al., 2011 |
| Reg1302R | AATTGCAAAGATCTATCCC |  |  |

**References:**

1. Hristov, A. N., Callaway, T. R., Lee, C. and Dowd, S. E. (2012). Rumen bacterial, archaeal, and fungal diversity of dairy cows in response to ingestion of lauric or myristic acid. J Anim Sci 90(12): 4449-4457.

2. Kittelmann, S., Seedorf, H., Walters, W. A., Clemente, J. C., Knight, R., Gordon, J. I. and Janssen, P. H. (2013). Simultaneous amplicon sequencing to explore co-occurrence patterns of bacterial, archaeal and eukaryotic microorganisms in rumen microbial communities. PLoS One 8(2): e47879.

**Table S2.** Primer sequences used for RT-PCR

| Primer’s  Name | Sequence  (5' to 3') | Product  Length (bp) | Primer  Efficiency (%) | Accession  Number |
| --- | --- | --- | --- | --- |
| TLRs (Toll like receptors) | | | | |
| *TLR1-F* | GCTGGCTGTTGCTGTGAAT | 128 | 99.2 | KF776538.1 |
| *TLR1-R* | AGTTCTTTGGCGTTCTTCCA |  |  |  |
| *TLR2-F* | GCCTCTCATCAGGCTTCTTC | 130 | 101.4 | DQ872435.1 |
| *TLR2-R* | TCTCGTTGTTGGACAGGTCA |  |  |  |
| *TLR3-F* | ATGGGCTGAAACAGACGAAT | 148 | 103.1 | HQ263210.1 |
| *TLR3-R* | AAAGAGCGAGAAGACAAATGC |  |  |  |
| *TLR4-F* | GGTTTCCACAAGAGCCGTAA | 187 | 97.2 | JF825527.1 |
| *TLR4-R* | GCGATAGAGTTCCACCTGCT |  |  |  |
| *TLR5-F* | CGATGCCTATTTGTGCTTCA | 186 | 99.1 | FJ659852.1 |
| *TLR5-R* | ACAGTCTTCCTGCTGCTCCA |  |  |  |
| *TLR6-F* | CTTTGTCCAGAGCGAGTGGT | 102 | 98.2 | HQ263211.1 |
| *TLR6-R* | ATGGGTTCCAGCAAGATCAG |  |  |  |
| *TLR7-F* | TGCGACATCTGGACCTCA | 138 | 102.8 | HQ263216.1 |
| *TLR7-R* | CAAACCACACAGCATCACAG |  |  |  |
| *TLR8-F* | GCAGAGGCTAATGGAGGAGA | 116 | 97.5 | HQ263212.1 |
| *TLR8-R* | GGAGGATGGAACTCTTGCAG |  |  |  |
| *TLR9-F* | GGGTCCTCAACCTCAAGTG | 181 | 98.4 | EU747825.1 |
| *TLR9-R* | AGCGACAGGGATACGAGAGA |  |  |  |
| *TLR10-F* | TGCCACAACAGAATCCAAGA | 179 | 101.6 | HQ263213.1 |
| *TLR10-R* | CCAGTCTCCACGCAAATAGG |  |  |  |
|  | | | | |
| Cytokines | | | | |
| *IL1α-F* | AATCTGGAGGAGGCAGTGAA | 138 | 99.8 | D63350.1 |
| *IL1α-R* | CTTTAGCAAGACGGGTTCGT |  |  |  |
| *IL1β-F* | AAGGCTCTCCACCTCCTCTC | 114 | 98.3 | DQ837160.1 |
| *IL1β-R* | TTGTCCCTGATACCCAAGG |  |  |  |
| *IL6-F* | TGACTTCTGCTTTCCCTACCC | 193 | 98.6 | HM565937.1 |
| *IL6-R* | GCCAGTGTCTCCTTGCTGTT |  |  |  |
| *IL10-F* | GCTGTTGCCTGGTCTTCCT | 178 | 103.5 | DQ837159.1 |
| *IL10-R* | TGTTCAGTTGGTCCTTCATTTG |  |  |  |
| *IL18-F* | ATTGCATCAGCTTTGTGGAA | 165 | 97.9 | AY605263.1 |
| *IL18-R* | CAGGTTGATTTCCCTGGCTA |  |  |  |
| *TNFα-F* | CCACTGACGGGCTTTACCT | 141 | 100.4 | AY304502.1 |
| *TNFα-R* | TGATGGCAGAGAGGATGTTG |  |  |  |
|  |  |  |  |  |
| TIR containing adaptors | | | | |
| *MYD88-F* | CGGCTGAAGTTGTGTGTGTC | 178 | 102.1 | JQ308783.1 |
| *MYD88-R* | CACCTGGAGAGAGGCTGAGT |  |  |  |
| *TICAM1-F* | GAAATCAGTCAGTCGCACACC | 139 | 104.2 | JQ863368.1 |
| *TICAM1-R* | TCATCTCCTCGGGTTCCTG |  |  |  |
| *TICAM2-F* | GAAGAAGACACAGCCGAAGC | 133 | 97.6 | JQ923482.1 |
| *TICAM2-R* | CGTTGACAGCATCATCCAAG |  |  |  |
|  | | | | |
| Tight junction proteins | | | | |
| *Occludin-F* | ACTACGCACCAAGCAATGAC | 198 | 98.3 | XM_005694596.2 |
| *Occludin-R* | AAGAGTGGAGGCAACACAGG |  |  |  |
| *Claudin1-F* | CAGCATGGTATGGCAATAGA | 183 | 98.6 | XM_005675123.2 |
| *Claudin1-R* | GCCTGGGTGTTGGGTAAG |  |  |  |
| *Claudin4-F* | CAACACCAGCAAGGAGGAAT | 194 | 102.3 | XM_005697785.1 |
| *Claudin4-R* | GAGCAGGGAGGATTGAAGAA |  |  |  |
|  |  |  |  |  |
| Internal reference | | | | |
| *GADPH-F* | TTCCACGGCACAGTCAAG | 116 | 99.8 | AJ431207.1 |
| *GADPH-R* | TACTCAGCACCAGCATCACC |  |  |  |
| *β-actin-F* | CTGGCACCACACCTTCTACA | 107 | 98.7 | JX046106.1 |
| *β-actin-R* | GGGTCATCTTCTCACGGTTG |  |  |  |

**Table S3.** Alpha diversity measures of each ruminal microbial domain for separation of different ages and feeding types in kids

| Microbial | Alpha index | Feeding pattern | Age (d) | | | | | SEM^*^ | *P* value^*^ | | | SEM^†^ | *P* for Age^†^ | |
| --- | --- | --- | --- | --- | --- | --- | --- | --- | --- | --- | --- | --- | --- | --- |
| domains |  |  | 0 | 7 | 28 | 42 | 70 |  | Type | Age | Type ×Age |  | L | Q |
| Bacteria | OTU number | S | 114 | 88.8 | 222.5 | 442 | 277 | 46.74 | <.0001 | 0.178 | 0.028 | 32.23 | <.0001 | 0.001 |
|  |  | G |  |  | 514.3 | 475 | 525.8 |  |  |  |  | 43.47 | <.0001 | 0.001 |
|  | Chao | S | 198.6 | 127.5 | 327.4 | 640.6 | 412.3 | 69.32 | 0.0002 | 0.235 | 0.03 | 45.27 | <.0001 | 0.001 |
|  |  | G |  |  | 742.5 | 673.4 | 754 |  |  |  |  | 65.55 | <.0001 | 0.002 |
|  | Ace | S | 267.3 | 149.5 | 394.1 | 674.5 | 477 | 86.98 | 0.005 | 0.498 | 0.103 | 68.46 | <.0001 | 0.02 |
|  |  | G |  |  | 751.5 | 676.5 | 806.2 |  |  |  |  |  |  |  |
|  | Shannon | S | 2.32 | 1.73 | 2.97 | 3.83 | 3 | 0.263 | 0.0002 | 0.28 | 0.154 | 0.251 | <.0001 | 0.007 |
|  |  | G |  |  | 4.31 | 4.24 | 4.34 |  |  |  |  |  |  |  |
|  | Simpson | S | 0.19 | 0.40 | 0.17 | 0.07 | 0.14 | 0.036 | 0.012 | 0.415 | 0.297 | 0.066 | 0.031 | 0.254 |
|  |  | G |  |  | 0.04 | 0.05 | 0.05 |  |  |  |  |  |  |  |
|  | Coverage | S | 1.000 | 1.000 | 0.990 | 0.980 | 0.990 | 0.002 | 0.0004 | 0.271 | 0.018 | 0.001 | <.0001 | 0.001 |
|  |  | G |  |  | 0.980 | 0.980 | 0.980 |  |  |  |  | 0.002 | <.0001 | 0.002 |
|  |  |  |  |  |  |  |  |  |  |  |  |  |  |  |
| Archaea | OTU number | S | - | 43.3 | 69.8 | 86.8 | 59.3 | 13.55 | 0.002 | 0.713 | 0.398 | 13.15 | 0.184 | 0.050 |
|  |  | G |  |  | 107.0 | 111.8 | 123.0 |  |  |  |  |  |  |  |
|  | Chao | S | - | 59.9 | 107.5 | 141.0 | 94.4 | 23.16 | 0.001 | 0.585 | 0.474 | 22.27 | 0.111 | 0.034 |
|  |  | G |  |  | 176.9 | 190.9 | 203.9 |  |  |  |  |  |  |  |
|  | Ace | S | - | 72.8 | 134.1 | 184.7 | 127.4 | 36.15 | 0.002 | 0.648 | 0.617 | 34.76 | 0.087 | 0.049 |
|  |  | G |  |  | 245.0 | 262.5 | 280.3 |  |  |  |  |  |  |  |
|  | Shannon | S | - | 1.10 | 2.12 | 1.66 | 0.98 | 0.165 | 0.007 | 0.044 | 0.001 | 0.216 | 0.348 | 0.003 |
|  |  | G |  |  | 2.01 | 1.74 | 2.30 |  |  |  |  | 0.159 | 0.001 | 0.233 |
|  | Simpson | S | - | 0.53 | 0.19 | 0.38 | 0.62 | 0.058 | 0.013 | 0.01 | 0.002 | 0.078 | 0.178 | 0.005 |
|  |  | G |  |  | 0.24 | 0.36 | 0.19 |  |  |  |  | 0.053 | 0.003 | 0.230 |
|  | Coverage | S | - | 0.997 | 0.996 | 0.994 | 0.996 | 0.001 | 0.001 | 0.413 | 0.571 | 0.001 | 0.104 | 0.060 |
|  |  | G |  |  | 0.992 | 0.991 | 0.990 |  |  |  |  |  |  |  |
|  |  |  |  |  |  |  |  |  |  |  |  |  |  |  |
| Fungi | OTU number | S | 78.7 | 50.0 | 42.7 | 75.0 | 52.3 | 21.23 | <.0001 | 0.787 | 0.485 | 18.86 | 0.699 | 0.794 |
|  |  | G |  |  | 150.0 | 142.3 | 167.7 |  |  |  |  | 17.74 | 0.001 | 0.155 |
|  | Chao | S | 105.5 | 66.2 | 61.2 | 109.3 | 68.5 | 31.12 | 0.0002 | 0.869 | 0.426 | 25.74 | 0.732 | 0.98 |
|  |  | G |  |  | 211.4 | 194.4 | 229.9 |  |  |  |  | 26.46 | 0.001 | 0.149 |
|  | Ace | S | 124.0 | 78.5 | 71.7 | 139.3 | 75.3 | 35.73 | 0.0004 | 0.878 | 0.221 | 26.16 | 0.649 | 0.742 |
|  |  | G |  |  | 236.4 | 203.7 | 260.2 |  |  |  |  | 32.1 | 0.002 | 0.309 |
|  | Shannon | S | 2.70 | 1.23 | 0.37 | 1.48 | 1.48 | 0.382 | <.0001 | 0.223 | 0.353 | 0.327 | 0.14 | 0.044 |
|  |  | G |  |  | 3.13 | 3.33 | 3.15 |  |  |  |  |  |  |  |
|  | Simpson | S | 0.12 | 0.56 | 0.87 | 0.54 | 0.44 | 0.106 | <.0001 | 0.168 | 0.13 | 0.09 | 0.168 | 0.047 |
|  |  | G |  |  | 0.10 | 0.07 | 0.13 |  |  |  |  |  |  |  |
|  | Coverage | S | 0.998 | 0.999 | 0.998 | 0.997 | 0.999 | 0.001 | 0.0001 | 0.81 | 0.476 | 0.001 | 0.17 | 0.207 |
|  |  | G |  |  | 0.995 | 0.996 | 0.995 |  |  |  |  |  |  |  |
|  |  |  |  |  |  |  |  |  |  |  |  |  |  |  |
| Protozoa | OTU number | S | - | - | 43.0 | 57.7 | 59.0 | 7.198 | 0.002 | 0.538 |  |  | 0.288 | 0.420 |
|  |  | G |  |  | 80.0 | 59.5 | 31.3 |  |  |  |  |  | 0.001 | 0.569 |
|  | Chao | S | - | - | 60.6 | 73.9 | 74.4 | 7.621 | 0.002 | 0.635 |  |  | 0.302 | 0.397 |
|  |  | G |  |  | 98.0 | 76.7 | 43.3 |  |  |  |  |  | 0.001 | 0.748 |
|  | Ace | S | - | - | 73.4 | 78.8 | 88.2 | 7.832 | 0.004 | 0.515 |  |  | 0.149 | 0.951 |
|  |  | G |  |  | 98.1 | 80.6 | 48.9 |  |  |  |  |  | 0.004 | 0.922 |
|  | Shannon | S | - | - | 1.33 | 1.65 | 1.29 | 0.284 | 0.373 | 0.861 |  |  | 0.46 | 0.458 |
|  |  | G |  |  | 1.83 | 1.4 | 1.17 |  |  |  |  |  |  |  |
|  | Simpson | S | - | - | 0.43 | 0.31 | 0.55 | 0.111 | 0.409 | 0.788 |  |  | 0.413 | 0.367 |
|  |  | G |  |  | 0.30 | 0.46 | 0.45 |  |  |  |  |  |  |  |
|  | Coverage | S | - | - | 0.999 | 0.998 | 0.999 | 0.0001 | 0.002 | 0.747 |  |  | 0.259 | 0.005 |
|  |  | G |  |  | 0.998 | 0.999 | 0.999 |  |  |  |  |  | 0.003 | 0.780 |

^*^SEM and *P* value for both groups from 28 to 70 d, fixed effects: feeding type, age, and feeding type × age interaction.

^†^SEM and *P* value for age (from 0 to 70 d), with age as the only fixed effect. L = linear effect of age, Q = quadratic effect of age.

S = Supplemental feeding; G = Grazing.
