# Supplementary material for: Shifts in Host Mucosal Innate Immune Function Are Associated with Ruminal Microbial Succession in Supplemental Feeding and Grazing Goats at Different Ages
Source: Front Microbiol. 2017 Aug 30;8:1655. doi: 10.3389/fmicb.2017.01655 (PMC5582421; doi:10.3389/fmicb.2017.01655)
Supplement: Supplementary file 3 [file Table_2.docx]

***Supplemental Text 1: Gene expression (RT-PCR analysis)***

Cloning was conducted using the primers listed in Table S2, and sequencing of corresponding recombinant plasmids (containing PCR products) verified that all the primers were specific to the corresponding genes. Standard curves were generated using the log_10_diluted both complementary DNA from pooling samples and recombinant plasmid. The results revealed that the amplification efficiency values were consistent between the target genes and the housekeeping gene (GADPH, glyceraldehyde-3-phosphate dehydrogenase) and β-actin, from 0.95 to 1.05.

Relative RT-PCR analysis was performed with the SYBR^®^ *Premix Ex Taq*^TM^ II (TliRNaseH Plus) detection kit (Takara, Japan) in the ABI 7900 Sequence Detection System. The assays were performed in a total volume of 10 μl, including 5 μl of SYBR® Premix Ex Taq™, 0.2 μl of ROX, 0.2 μl of each primer (10 μM), 3.4μl of RNase-free water and 1 μl of cDNA sample. The SYBR Green 2-step PCR protocol was as follows: 95°C for 30 s, forty cycles of 95°C for 5 s and 60°C for 30 s. A melt curve was generated at 95°C for 15 s, 60°C for 30 s, and 95°C for 15 s. All the samples were analysed in triplicate, and relative gene expression data were calculated using the 2^-△△Ct^ method.

***Supplemental Text 2: OTU diversity and similarity analysis***

Average sequence number of bacteria, archaea, fungi and protozoa were 37017, 64250, 103886, and 65374, respectively, with the least sequence number reached 10819, 6004, 10412, 11241, respectively. Even at the least sequence number, the coverage for microbiota reached 0.98 to 1.00, suggesting our sampling depth was enough (Table S3). The classified OTU number for bacteria, archaea, protozoa and fungi were 2925,1949, 910 and 252, respectively.

Alpha diversity of ruminal bacteria, archaea and fungi was more abundant (*P* < 0.05) in G kids compared to that in S kids. Alpha diversity indices (OTU number, Chao, Ace and Shannon) of ruminal bacteria and archaea in both groups increased with age (*P* < 0.01). For G kids, alpha diversity indices of ruminal fungi increased (*P* < 0.01), while those indices of ruminal protozoa decreased quadratically (*P* < 0.05) with age. PCoA analysis revealed that microbial communities could be clearly discriminated by both age and feeding type (Fig. 1).

tb-PCA revealed the top 10 OTUs of each microbial domain contributing to separation of different ages and feeding types (Fig. 2). For bacteria, two OTUs (OTU13 and 129), and four OTUs (OTU5, 15, 102 and 977) accounted for separation of d 0 and 7 from others (Fig.2A). Three OTUs (OTU76, 144 and 246) could separate G from S kids (Fig.2B). For archaea, four OTUs (OTU1, 7, 13 and 17) contributed to discriminate d 7 from others (Fig.2C). OTU5 contributed most for separation G from S kids (Fig.2D). For fungi and protozoa, no OTUs can clearly discriminate different ages (Fig. 2E and 2G). Three OTUs (OTU391, 552 and 626) of fungi account for separation of S kids from others (Fig. 2F), and four OTUs (OTU40, 59, 97 and 250) account for separation of most G kids (Fig. 2H).
